# Exploring Radiation Response in Two Head and Neck Squamous Carcinoma Cell Lines Through Metabolic Profiling

**DOI:** 10.3389/fonc.2019.00825

**Published:** 2019-08-30

**Authors:** Eva Lindell Jonsson, Ida Erngren, Mikael Engskog, Jakob Haglöf, Torbjörn Arvidsson, Mikael Hedeland, Curt Petterson, Göran Laurell, Marika Nestor

**Affiliations:** ^1^Department of Surgical Sciences, Uppsala University, Uppsala, Sweden; ^2^Department of Medicinal Chemistry, Uppsala University, Uppsala, Sweden; ^3^Medical Product Agency, Uppsala, Sweden; ^4^Department of Immunology, Genetics and Pathology, Uppsala University, Uppsala, Sweden

**Keywords:** radioresistance, radiosensitivity, metabolomics, mass spectrometry, redox status

## Abstract

Head and neck squamous cell carcinoma (HNSCC) is the sixth most common form of cancer worldwide. Radiotherapy, with or without surgery, represents the major approach to curative treatment. However, not all tumors are equally sensitive to irradiation. It is therefore of interest to apply newer system biology approaches (e.g., metabolic profiling) in squamous cancer cells with different radiosensitivities in order to provide new insights on the mechanisms of radiation response. In this study, two cultured HNSCC cell lines from the same donor, UM-SCC-74A and UM-SCC-74B, were first genotyped using Short Tandem Repeat (STR), and assessed for radiation response by the means of clonogenic survival and growth inhibition assays. Thereafter, cells were cultured, irradiated and collected for subsequent metabolic profiling analyses using liquid chromatography-mass spectrometry (LC-MS). STR verified the similarity of UM-SCC-74A and UM-SCC-74B cells, and three independent assays proved UM-SCC-74B to be clearly more radioresistant than UM-SCC-74A. The LC-MS metabolic profiling demonstrated significant differences in the intracellular metabolome of the two cell lines before irradiation, as well as significant alterations after irradiation. The most important differences between the two cell lines before irradiation were connected to nicotinic acid and nicotinamide metabolism and purine metabolism. In the more radiosensitive UM-SCC-74A cells, the most significant alterations after irradiation were linked to tryptophan metabolism. In the more radioresistant UM-SCC-74B cells, the major alterations after irradiation were connected to nicotinic acid and nicotinamide metabolism, purine metabolism, the methionine cycle as well as the serine, and glycine metabolism. The data suggest that the more radioresistant cell line UM-SCC-74B altered the metabolism to control redox-status, manage DNA-repair, and change DNA methylation after irradiation. This provides new insights on the mechanisms of radiation response, which may aid future identification of biomarkers associated with radioresistance of cancer cells.

## Introduction

Every year more than half a million new cases of squamous cell carcinoma of the head and neck (HNSCC) are reported (1), which makes it the 6th most common type of cancer worldwide (2). HNSCC is a heterogeneous disease, including epithelial cancers of the oral cavity, lip, nasal cavity, paranasal sinuses, larynx, pharynx, and salivary glands. Despite the high frequency of HNSCC worldwide, it has one of the lowest survival rates among the more common cancer types (3), and almost 50% of all patients with HNSCC will die from their disease (4). Treatment challenges include complex anatomy, functional preservation of substantial organs, and minimization of side effects (5).

Radiotherapy (RT), the clinical application of ionizing radiation, is one of the most effective tools in therapy of cancer today (6). Even though advances in treatment methods have been made during recent decades, RT with or without surgery remains the major approach to curative treatment of HNSCC (7). The efficacy of RT is however still limited by different technological, biological, and clinical constraints. HNSCC is on average only moderately radiosensitive, which means that radiotherapy often must be given to such an extent that it approaches the maximum tolerated dose for the surrounding normal tissue. This may cause substantial acute and late toxicities, resulting in significant morbidity and altered quality of life (8). Moreover, the individual heterogeneity in terms of HNSCC radiosensitivity is immense, and therapeutic responses to RT have been shown to vary from complete to no response (9, 10). Consequently, there is a great need for individualized radiotherapy treatment approaches in HNSCC, to aid in predicting and monitoring tumor response to radiotherapy before, during, and after treatment. This requires new insights on the mechanisms of radiation response, novel markers to predict tumor response to radiotherapy, as well as potential treatment targets to enhance radiation-sensitivity.

The metabolism of cancer cells differs from normal differentiated cells (11). Tumor progression, the development of increasingly aggressive and resistant tumor cells, has increasingly been understood to be associated with perturbations in cellular metabolism, such as increased glutaminolysis and fatty acid oxidation, the Warburg effect, as well as altered patterns of macromolecule synthesis and storage (11). Cancer cells are able to adapt metabolically to many types of cellular stress, for example, hypoxia, nutrient depletion, and radiation, and studies have shown several mechanistic links between cellular metabolism and growth control (11). No doubt, the plasticity of cancer cell metabolism can be vital for causing many patients to relapse into disseminated and treatment-resistant disease.

Cancer metabolism is altered by ionizing radiation. Radiation exposure induces different types of genome damage, including DNA single- and double-strand breaks and bulky lesions. Thereby multiple signaling pathways are activated, involved in e.g., DNA damage response, signal transduction pathways, and regulation of survival (9, 12, 13). Enzymatic pathways quickly repair many of these lesions, but lesions that are not repaired correctly lead to chromosomal abnormalities and a possible change of cell phenotype, culminating in cell cycle arrest and/or cell death. In addition to rapid proliferation, many cancer cells are also deficient in repair proteins and cell cycle checkpoints, making them more sensitive to radiation (12). Cellular exposure to ionizing radiation also triggers a complex series of molecular responses that can affect metabolism, either directly or indirectly, by altering cell growth (14). Ionizing radiation also impacts multiple cellular compartments even at relatively modest doses, which can also trigger a variety of signaling pathways (15). These molecular events are not only important for the therapeutic response, but may also influence the inflammatory response at a local and systemic host level. Subsequently, ionizing radiation will result in different alterations in the metabolome, depending on what pathways and processes that the specific cell alters in response to radiation.

Several studies have identified different molecular entities associated with radioresistance, nevertheless the underlying mechanisms are still inconclusive (16). Suggested mechanisms include hypoxia, alterations of the DNA damage response, activation of pathways involved in pro-survival or cell cycle regulation, as well as vascular, stromal, and immunological changes (17–26). However, a majority of these studies are based on genome, transcriptome, and proteome data. Consequently, it would be of major interest to conduct studies in this field closer to the phenotype, such as metabolite profiling (27). Metabolomics in radiation biology have previously been used for two main purposes (i) metabolic profiling for utilization in biodosimetry or for biomarker discovery of radiation exposure (14, 28–39) and (ii) metabolic profiling for a more mechanistic understating of the radiation response of the metabolome (14, 34, 40–43) However, to date no study has focused on investigating the different metabolic responses of genetically similar cells with divergent radiation sensitivities.

Metabolic profiling is the comprehensive and quantitative analysis of small endogenous metabolites that are the downstream products in biological systems. It can be a powerful approach to study the phenotype of cancer cells, since it analyses the biochemical outcome of the activities of the proteome (27). The promise of metabolomics as a scientific tool has been fueled largely by the advancement in nuclear magnetic resonance (NMR) and mass spectrometry (MS), and could be a well-suited and cost-effective complement to current genomic and proteomic data in the field (14).

Even though our current understanding of the mechanisms in play during radiation of tumor cells, and how they are related to radiosensitivity, is incomplete (40), it has been shown to at least in part be due to the different metabolic alterations that the tumor can make (44). Ionizing radiation induces complex biological responses that interfere with gene and protein expression, which disrupts normal metabolic processes in cells and organs. As a result, metabolites related to classical pathways of radiation damage, including oxidative stress and subsequent DNA breakdown have been shown to be affected. Additionally, polyunsaturated fatty acids (PUFA) metabolism can be disrupted as an inflammatory effect of radiation exposure (14). Changes in nicotinate and nicotinamide metabolism and cofactor biosynthesis have also been reported in radiation related research, suggested to be linked to DNA repair (14, 45, 46).

Consequently, metabolomics may provide insights into the mechanism behind a reduced sensitivity to radiotherapy by identifying differences in metabolites produced in response of irradiation in cancer cells with different sensitivity to radiotherapy. This may provide significant mechanistic understanding related to cellular response due to perturbations caused by radiation treatment (14), which might be a possible way to find a pharmacologically alterable pathway that is altered in the less sensitive cells, or to predict response or non-response to radiation therapy.

In the present study, we investigate the relationship between radiation response and the metabolome of HNSCC in a unique model system, using two HNSCC cell lines from the same donor but with different radiosensitivity. This enables the study of their metabolic response to radiation in an exceptionally controlled setting, and has to our knowledge not been performed previously. The aim was to assess the metabolic differences between the two cell lines, and how this was affected by radiation. Cells were first genotyped, and assessed for radiosensitivity using clonogenic survival and long-term growth inhibition assays at several radiation doses. Cell-based metabolic profiling using liquid chromatography hyphenated to high resolution mass spectrometry (LC-HRMS) was then performed to investigate the influence of early and intermediate radiation responses on metabolites, and to assess the potential correlation to the different radiosensitivities of the cells. In the long-term, the study may contribute to provide new insights on the mechanisms of radiation response, discover possible biomarkers for radiation-sensitivity, and possibly present potential treatment targets in order to enhance radiation-sensitivity of HNSCC.

## Materials and Methods

This study has been conducted in accordance with Frontiers guidelines on study ethics. It does not involve any animal or human subjects or identifiable human data, thus does not require ethical permission.

### Cell Lines

The squamous cell carcinoma cell lines UM-SCC-74A and UM-SCC-74B were kindly provided by Professor TE Carey (University of Michigan, USA), and have previously been described by Brenner et al. (47). The cell lines were taken from the same male patient with oral squamous cell carcinoma after radiochemotherapy (UM-SCC-74A) and then again at surgery for persistent cancer (UM-SCC-74B) (47). Cells were cultured at 37°C, in 5% CO_2_ in DMEM medium containing 2 mM l-glutamine (Biochrom GmbH, Germany), supplemented with 5% fetal bovine serum (Sigma-Aldrich, Germany), MEM non-essential amino acids (Sigma-Aldrich AB, Germany), and antibiotics (100 IU penicillin and 100 μg/ml streptomycin) (Biochrome GmbH, Germany).

### Genotyping

UM-SCC-74A and UM-SCC-74B cells were Genotyped using Short Tandem Repeat (STR) Analysis in order to verify the origin and similarity of the cell lines. DNA was extracted from frozen cell cultures and analyzed using the AmpFLSTR® Identifiler® PCR Amplification Kit. The Identifiler kit amplifies 15 loci and Amelogenin in a single tube and provides loci consistent with major worldwide STR databasing standards.

### Clonogenic Survival

Clonogenic survival assays were performed as described previously (48), in order to assess the radiosensitivity of UM-SCC-74A and UM-SCC-74B cells. In short, cells were pre-plated into 25 cm^2^ culture flasks with 8 ml complete medium. After 48 h, cells were exposed to external beam radiation using ^137^Cs gamma-ray photons at a dose-rate of ~1 Gy/min (Best Theratronics Gammacell® 40 Exactor, Springfield, USA), corresponding to a dose of 0, 2, 4, 6, or 8 Gy. Colonies were allowed to form for 10–14 days. Cells were then fixated with 95% ethanol and stained with crystal violet. The colonies were inspected under a microscope, and only cells giving rise to colonies consisting of 50 or more cells were considered clonogenic survivors. Plating efficiency, PE (number of colonies formed/number of cells seeded in the control), and the survival fraction (number of colonies formed after treatment/number of cells seeded × PE) were calculated in Microsoft Office Excel 2016 for Mac version 14.6.1 (Microsoft, Redmond, WA, USA). Statistical analyses were performed using GraphPad Prism 6 (GraphPad Software, San Diego, CA, USA). Differences in normalized survival fractions of 2 Gy irradiated UM-SCC-74A cells vs. UM-SCC-74B cells were assessed using an unpaired *t*-test and were considered statistically significant if *P* < 0.05.

### Radiation Induced Long-Term Growth Inhibition

As a complement to the clonogenic- and 3D cell culture assays, the long-term growth inhibitory effects of radiation were evaluated using a growth inhibition assay as described earlier (49). In short, UM-SCC-74A or UM-SCC-74B cells were pre-plated into 25 cm^2^ culture flasks with complete medium. After 48 h, cells were exposed to external beam radiation corresponding to a dose of 0, 2, 4, 6, or 8 Gy. Cells were then counted and reseeded about once a week, and the corresponding total cell numbers were calculated. The increase in cell number was followed for 4 weeks. Cell doubling times were calculated using the least square fitting method. In order to determine any statistically significant differences from the untreated group at the last data point, total cell numbers were analyzed using one-way ANOVA followed by Dunnett's multiple comparisons test in GraphPad Prism and were considered statistically significant if *P* < 0.05.

### Radiation Response in 3D Cell Culture

For liquid overlay 3D multicellular tumor spheroid formation, 96-well plates were coated with 0.15% agarose dissolved in PBS with 1% penicillin/streptomycin. One thousand UM-SCC-74B cells/well or 1500 UM-SCC-74A cells/well were seeded and incubated at 37°C in supplemented media for 3 days prior to irradiation with 2 Gy or mock radiation (0 Gy) using ^137^Cs gamma-ray photons as described above. Spheroid images were obtained at start of treatment and 10 days after treatment using a Canon EOS 700D camera mounted on an inverted Nikon Diaphot-TMD microscope. The images were analyzed using ImageJ version 1.48 (NIH, Bethesda, MD, USA), by measuring the surface area of each spheroid and calculating the volume, assuming each spheroid retained a spherical form. Each spheroid was normalized to its own starting volume at the start of treatment (Day 0, growth ratio = 1). Statistical analyses were performed using GraphPad Prism 6 (GraphPad Software, San Diego, CA, USA). Differences in normalized spheroid growth ratios of UM-SCC-74A cells vs. UM-SCC-74B cells were assessed using an unpaired *t*-test and were considered statistically significant if *P* < 0.05.

### Measurement of Cleaved Poly ADP Ribose Polymerase (PARP)

Levels of cleaved PARP1 in cell lines were measured using ELISA. The assay detects the presence of the 89 kDa PARP1 fragment containing the catalytic domain. The proteolysis of PARP1 by activated caspase-3 renders the enzyme inactive, which further facilitates apoptotic cell death. Thus, the presence of the 89 kDa PARP1 fragment is considered to be a reliable biomarker of apoptosis. Cells were incubated for 48 h prior to irradiation (2 Gy) or mock radiation (0 Gy) using ^137^Cs gamma-ray photons as described above. Whole-cell lysates were prepared 12 h after irradiation according to standard protocols. Cell lysates were diluted 1:1,000. The Cleaved PARP1 Human SimpleStep ELISA^®^ Kit (Abcam, Cambridge, UK) was used according to the manufacturer's protocol. The OD was then measured at 450 nm using a microtiter plate reader (BioRad, USA). Statistical analyses were performed using GraphPad Prism 6.

Differences in cleaved PARP1 levels were assessed using an unpaired *t*-test and were considered statistically significant if *P* < 0.05.

### Irradiation of Cells for Metabolic Profiling

Two days before irradiation (18–25) × 10^6^ UM-SCC-74A or (10–25) × 10^6^ UM-SCC-74B cells were cultured in cell culture plates (*n* = 46 and *n* = 52, respectively, Nunclon Surface, 15 cm diameter, Cat No. 168 381, 145 cm^2^) at 37°C, in 5% CO_2_ in supplemented DMEM medium. At the time of irradiation, cells were exposed to external beam radiation corresponding to a dose of 0 or 2 Gy. Cells were subsequently harvested at ~75% confluence at 4 h (*n* = 20 and 22 for UM-SCC-74A and UM-SCC-74B, respectively) and 24 h (*n* = 26 and 30 for UM-SCC-74A and UM-SCC-74B, respectively) after irradiation as according to Engskog et al. (50). The time points were chosen in order to detect IR-induced perturbations in the cell metabolome in the most optimal settings possible. These time points have been shown to be relevant for IR-induced early- and intermediate cellular responses in previous cell-based radiation metabolomic assessments (40, 41, 43). Moreover, while the cellular responses have occurred at these time points, they have not yet resulted in apoptosis in the majority of cells. Consequently, in these experimental settings a majority of the irradiated cells can be harvested, reducing any risk of selection-biased analyses. All cell sample harvesting was performed on ice. Cell medium was removed and cells were rapidly washed three times with cold, sterile phosphate buffered saline (PBS, Medicago, Uppsala, Sweden), followed by detachment of cells using a 23 cm long rubber-tipped Nunc cell scraper (Thermo Scientific). The detached cells were collected in 3.5 ml cold MilliQ water, transferred to 15 mL polypropylene brown tubes (Greiner bio-one GmbH, Germany) and snap-frozen in liquid N_2_ followed by thawing at 37°C for 10 min. The freeze/thaw cycle was then repeated twice with subsequent sonication on ice for 30 s. Samples were stored at −80°C until metabolite extraction.

### Metabolite Extraction

Prior to extraction of the intracellular metabolites, the samples were randomized into five separate sample batches comprised of 20 samples each. Each sample batch was prepared and analyzed separately. The samples were thawed at room temperature and centrifuged at 2000 RCF for 10 min at 4°C. A quality control (QC) sample was created for each batch by pooling an equal volume from all samples within each batch. The QC sample was extracted as described below. The five QC samples were pooled after extraction of all five batches. The aqueous supernatants were transferred to fresh extraction tubes followed by addition of chloroform and methanol for the final proportion 2.85:4:4 water:methanol:chloroform (51–53). The samples were vortexed gently and stored at 8°C for 20 min. to the samples were the centrifuged for 20 min at 2000 RCF and 4°C. The aqueous phases were recovered and evaporated to dryness at 40°C under a gentle stream of N_2_(g).The samples were stored at −80°C after evaporation. Prior to analysis the samples were reconstituted in acetonitrile:Milli-Q water 76:24.

### Metabolite Profiling With LC-MS

All metabolite profiling analyses were performed on an Acquity UPLC I-class system from Waters (Manchester, UK) hyphenated to a G2S Synapt Q-TOF equipped with an electrospray ionization (ESI) source (Waters). All systems were controlled using Masslynx version 4.1 (Waters). For chromatographic sample separation prior to detection a Acquity BEH amide column (1.7 μm, i.d. 2.1 × 50 mm) from Waters was used. The column temperature was kept at 40°C for all analyses and the injection volume was 5 μl. Mobile phase A consisted of 95:5 acetonitrile/water with 10 mM ammonium formate and 0.1% FA and mobile phase B consisted of 50:50 acetonitrile/water with 10 mM ammonium formate and 0.1% FA. A non-linear elution gradient from 100% A to 100% B was used, the flow rate was set to 0.3 ml/min. In detail: 100% A was kept for 0.5 min then decreased non-linearly (slope-factor 8 in MassLynx) over 12.5 min to 100% B, 100% B was held for 4 min followed by 6 min at 100% A to re-equilibrate the column for a total run-time of 23 min. Detection was performed in both positive and negative ionization mode in resolution MS^E^-mode within the scan-range *m/z* 50–800. All samples were analyzed in negative ionization mode first, followed by positive ionization mode. The capillary voltage was 1 and −2 kV and the cone voltage was set to 30 and 25 V in positive and negative ionization mode, respectively. In both ionization modes the source temperature was 120°C. Nitrogen was used as desolvation gas at the flow-rate 800 l/h and the desolvation temperature was 500°C and 450°C in positive and negative mode, respectively. Nitrogen was used as cone gas as well at a flow-rate of 50 l/h. and. A collision energy ramp from 20 to 45 eV was used for MS^E^ acquisition with argon as collision gas. Lock-mass correction for accurate *m/z* measurements was applied using a solution of leucine-enkephalin (*m/z* 556.2766). Each sample batch was analyzed separately, i.e., one sample batch per day. Prior to each sample batch analysis the instrument was mass calibrated and the sample cone was cleaned. A reference mix (30 μM of hypoxanthine, cytidine, phenylalanine, tryptophan, and glutamine, respectively) was analyzed before and after each batch to check the system suitability with regard to mass accuracy, instrument sensitivity, and column performance. The QC sample was analyzed repeatedly prior to the study samples for system conditioning, to ensure stable analytical conditions, as well as, between the randomized study samples in regular intervals to monitor the analytical stability throughout the analysis (54).

### Chemicals

Formic acid, FA(LC-MS grade), ammonium formate (LC-MS grade), methanol (LC-MS grade), Cytidine (99%), hypoxanthine (≥99%), and tryptophan (≥99.5%) were purchased from Sigma Aldrich (Steinheim, Germany). Phenylalanine (>99%) was purchased from MERCK (Kenilwoth, N.J., USA) while glutamine (>99%) was purchased from Fluka (Buchs, Switzerland). Acetonitrile (LC-MS grade) was obtained from Fischer Scientific (Zurich, Switzerland) and chloroform (analytical grade) was purchased from BDH Laboratory Supplies (Poole, England, UK). Leucine-encephalin was prepared and certified by ERA (Golden, CO, USA). The water was purified using a Milli-Q™ water system from MilliPore (Bedford, MA, USA).

### Data Processing

Data quality was assessed through in-depth examinations of five representative metabolites spread out in the obtained chromatograms; hypoxanthine, cytidine, phenylalanine, tryptophan, and glutamine. The evaluation was performed by univariate data analysis based on all QC sample injections, in total 25 injections (20% of all sample injections), prior to data pre-processing and multivariate data analysis. Mass accuracy, retention time, and peak area was monitored to check system stability throughout the analysis. DataBridge (Masslynx version 4.1, Waters) was used to convert the raw data files to NetCDF files. The R-based software XCMS was used for peak detection, retention time alignment, and peak grouping (55). The centWave function was used for peak detection with the following function parameters; the maximal deviation in m/z between scans was set to 10 ppm, the boundaries for peak width was set between 5 and 45 s and the signal to noise ratio cut-off was set to 5. The “obiwarp” function was used for retention time alignment. The processed data was subjected to adduct, isotope and fragment annotation using the R-Package, CAMERA (56). The resulting dataset was exported to Microsoft Excel and prior to normalization all features with a retention time <50 s were removed. The data was normalized using LocalMean correction where all features were normalized to the feature mean of the QC:s in the respective batches (57). After normalization, all features with coefficient of variance (CV) >30% in the QC samples were removed (54, 58, 59).

### Multivariate and Univariate Data Analysis

The reduced and filtered data sets from the data processing were analyzed by multivariate data analysis using SIMCA-P+ (version 14, Umetrics, Umeå, Sweden). All data was pareto scaled prior to multivariate data analysis. Principal Component Analysis (PCA) was used to find sample clustering, identify possible sample outliers, and systematic trends in the data. Orthogonal Projection to Latent Structures- Discriminant Analysis (OPLS-DA) in combination with S-Plots were used analyse differences between sample groups and to identify differentiating features between sample groups (60, 61).

Comparisons were made between the two cell line controls as well as between irradiated cells and controls of the respective SCC cell lines. The irradiated cells were further divided into subgroups of rapid response and intermediate response depending on the time between cell irradiation and cell sample harvesting. Rapid response subgroups were harvested 4 h after irradiation and intermediate response subgroups were harvested 24 h after irradiation.

Features with *p*-corr values >0.4 were selected and annotated. Molecular weight, isotopic patterns, fragmentation and, when possible, retention time comparison to an in-house database were utilized for feature annotation. The Human Metabolome Database (HMDB), METLIN and in-house databases was utilized to search for the experimental *m/z* values with a molecular weight difference tolerance of 30 ppm. The raw data signal isotopic pattern, fragmentation (when reference spectra was available) as well as related adducts present at the same retention time in the raw data were all matched against the plausible hits from the data base search. All annotated metabolites should be considered putatively annotated (level 2) according to the Metabolomics Standards Initiative nomenclature (62). All annotated metabolites were subjected to pathway analysis using MetaboAnalyst 3.0 and the highest score pathways were subjected to further data analysis. One-Way Analysis of Variance (ANOVA) and *post-hoc* Tukey tests using Origin 2015 (OriginLab corporation, Northampton, MA, USA) was used for univariate significance testing of all annotated features and *p*-values <0.05 was considered significant. The significantly altered metabolite are presented as fold changes with 95% confidence intervals. The confidence intervals of fold changes were calculated using Fieller's theorem.

## Results

### Genotyping

STR results demonstrated identical results for unirradiated UM-SCC-74A and UM-SCC-74B (Supplemental Table 1), verifying the origin and similarity of UM-SCC-74A and UM-SCC-74B cells.

### Clonogenic Survival, Cell Growth, and Apoptosis Assays

**Figure 1:**
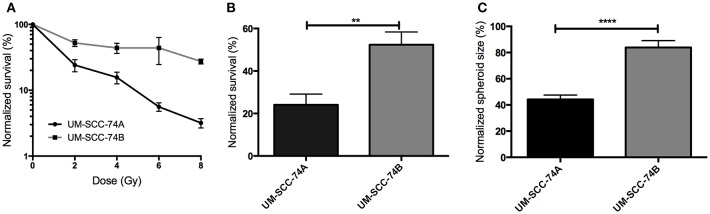
**(A)** Clonogenic survival assay of UM-SCC-74A and UM-SCC-74B cells treated with a radiation dose of 0, 2, 4, 6, and 8 Gy. *N* > 3. Groups are normalized to the plating efficiency of the non-irradiated controls. Error bars represent the standard error of mean. **(B)** Comparison of clonogenic survival of UM-SCC-74A and UM-SCC-74B cells treated with a radiation dose of 2 Gy. *N* > 6. Difference in survival fraction was assessed using an unpaired *t*-test. **p < 0.01. **(C)** Assessment of UM-SCC-74A and UM-SCC-74B spheroid growth 10 days after 2 Gy irradiation. Groups are normalized to the growth ratio of the non-irradiated controls. N > 5. Difference in spheroid growth was assessed using an unpaired *t*-test. ****p < 0.0001.

**Figure 2:**
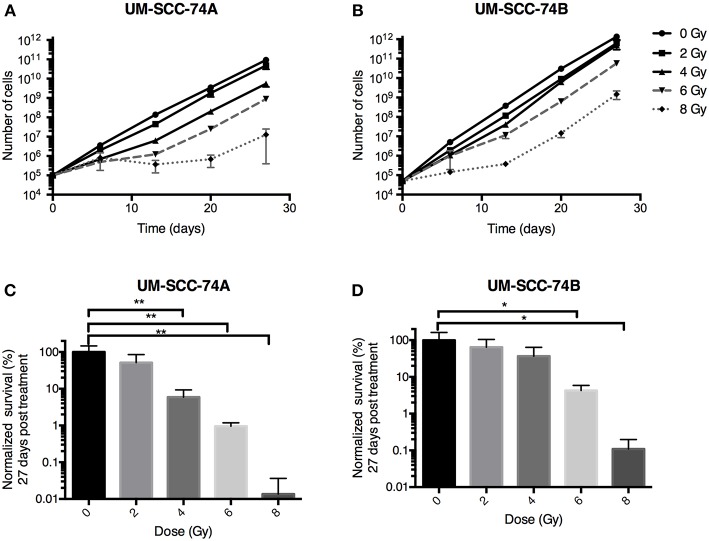
Growth inhibition studies of UM-SCC-74A and UM-SCC-74B cells treated with radiation (0, 2, 4, 6, or 8 Gy). Error bars represent the standard error of mean. *N* = 3. **(A,B)** Cell growth followed over time. **(C,D)** Comparison at day 27. Differences in total cell numbers from control at day 27 were analyzed using one-way ANOVA followed by Dunnett's multiple comparisons test. *p < 0.05, **p < 0.01.

The effect of radiation on UM-SCC-74A and UM-SCC-74B cell viability was studied using clonogenic survival assays, 3D cell growth assays, and long term growth inhibition assays, and can be seen in Figures 1, 2. In all three assays, UM-SCC-74A cells proved more sensitive to radiation than UM-SCC-74B cells.

In the clonogenic survival assay (Figures 1A,B), UM-SCC-74A cells demonstrated a lower survival fraction than UM-SCC74B at all radiation doses assessed (Figure 1A). Accordingly, a radiation dose of 2 Gy to UM-SCC-74A resulted in a survival fraction of 24 ± 5 (SEM)% of the unirradiated controls, whereas the survival fraction of irradiated UM-SCC-74B cells was significantly higher, 52 ± 6% (Figure 1B).

In the three-dimensional cell growth assay (Figure 1C), spheroid growth was followed for 10 days after irradiation, reflecting effects of both cell death and growth inhibition in a more *in vivo*-like environment (63). Also here, UM-SCC-74B cells were significantly less affected by radiation, where a radiation dose of 2 Gy resulted in a normalized spheroid size of 84 ± 5 (SEM)% of unirradiated controls, compared to 44 ± 3% for UM-SCC-74A spheroids.

In the long-term cell growth assay, cell growth was followed for 4 weeks' time, reflecting long-term effects of both cell death and growth inhibition. Doubling times of unirradiated UM-SCC-74A and UM-SCC-74B cells were 1.48 and 1.28 days, respectively. Also in this assay, UM-SCC-74A cells were more affected by radiation than UM-SCC-74B cells (Figures 2A,B). At the last assessed time point (27 days), UM-SCC-74A cells exposed to 2 Gy of irradiation were 52 ± 19% (SEM) of unirradiated controls. For 4, 6, and 8 Gy, the corresponding numbers were 5.9 ± 2.0, 1.0 ± 0.1, and 0.01 ± 0.01% (Figure 2C). For UM-SCC-74B cells, 2 Gy irradiation resulted in a cell number of 65 ± 12% of unirradiated controls at day 27, and for 4, 6, and 8 Gy the corresponding numbers were 37 ± 16, 4.2 ± 0.9, and 0.11 ± 0.05% (Figure 2D).

Moreover, apoptosis was also studied in the cells using a cleaved PARP1 assay (Supplemental Figure 1). Levels of cleaved PARP1 were significantly increased in 2 Gy irradiated UM-SCC-74A cells compared to unirradiated cells, whereas levels did not significantly differ for UM-SCC-74B cells.

Consequently, as all these independent assays demonstrated UM-SCC-74A cells to be clearly more affected by radiation in terms of cell viability and growth than UM-SCC-74B cells, UM-SCC-74A are referred to as “radiosensitive” and UM-SCC-74B cells as “radioresistant” in the present study. These are to be viewed as relative terms, where UM-SCC-74A cells are “radiosensitive” in relation to UM-SCC-74B cells and vice versa.

### Metabolic Profiling Data Quality Control

Mass accuracy, retention time, and peak area of five metabolites; hypoxanthine, cytidine, phenylalanine, tryptophan, and glutamine were monitored in the QC samples throughout the analysis to verify system stability. The mass error was found to be within 10 and 12 ppm in positive and negative ionization mode, respectively, and the variation in retention time displayed a CV-value below 1.5% throughout the analysis. The peak areas over the five analytical batches evidenced, as expected, some batch variations; this was however corrected for after the normalization by local mean correction as no sample separation due to batch effects were found in the multivariate data analysis. The peak areas of hypoxanthine, cytidine, phenylalanine and tryptophan varied from 26 to 48% (CV) in the raw data while glutamine showed huge variations of up to 128%.

### Metabolic Profiling

**Figure 3:**
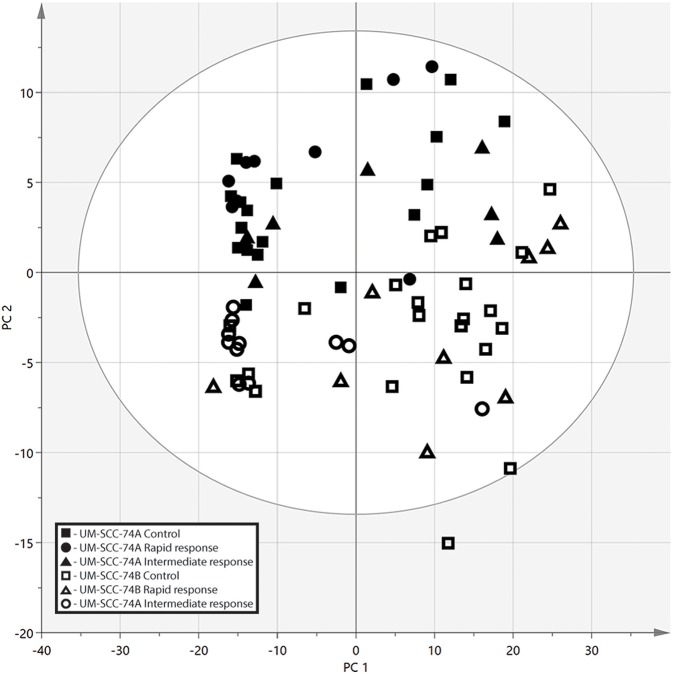
PCA score plot from the metabolic profiling in negative ionization mode, the two SCC cell lines are separated along the second principal component (PC2). Along the first component (PC1) there is some separation between the controls and irradiated cells, however, no clear clustering. UM-SCC-74A Control (■), UM-SCC-74A Rapid response (4 h after 2 Gy irradiation) (▴), UM-SCC-74A Intermediate response (24 h after 2 Gy irradiation) (∙), UM-SCC-74B Control (□), UM-SCC-74B Rapid response (▵), and UM-SCC-74B Intermediate response (°).

Multivariate data analysis was performed on the pre-processed and filtered data using PCA and OPLS-DA. All samples were analyzed with the unsupervised model PCA to examine possible sample group separations and sample clustering behavior. The PCA scores plot revealed clear discrimination between the intracellular metabolome of the SCC cell lines in the second component (PC2) (Figure 3). There was some separation between irradiated cells and controls along the first component (PC1), the separation was most pronounced for the less radiosensitive UM-SCC-74B cell line. However, for both cell lines the controls cluster at the left hand side of the scores plot with the irradiated cells on the right hand side.

The supervised model OPLS-DA was used to analyse the metabolic changes due to irradiation in the two SCC cell lines as well as the metabolic differences between the SCC cell line controls (unirradiated controls). The metabolic changes after irradiation in each SCC cell line was separated into rapid response (cells were harvested 4 h after irradiation) and intermediate response (cells were harvested 24 h after irradiation), respectively.

The metabolic profiling demonstrated a number of significant (*p* < 0.05) metabolic differences between the two non-irradiated cell line controls (Table 1). Several metabolites connected to nicotinate and nicotinamide metabolism such as nicotinic acid, nicotinamide, and nicotinic acid mononucleotide were found altered in the UM-SCC-74B cell line as compared to UM-SCC-74A (*p* = 0.005). Moreover, guanosine, inosine, xanthine, and the purine intermediate 5-phosphoribosylamine were found at significantly different levels in the two cell lines indicating changes in purine metabolism and biosynthesis between UM-SCC-74A and UM-SCC-74B (*p* = 0.006).

**Table 1:** Significantly different metabolites (*p* < 0.05) between non-irradiated cell lines UM-SCC-74A and UM-SCC-74B are presented as fold changes (95% confidence).

**POSITIVE IONIZATION**	
**Metabolite ID**	**A control vs. B control**
5-phosphoribosylamine	−2.10 ± 0.66
DMAPP	2.39 ± 1.20
**NEGATIVE IONIZATION**	
**Metabolite**	**A control vs. B control**
Tyrosine	−2.01 ± 0.74
Valine	−3.35 ± 1.72
Alanine, sarcosine, beta-alanine	−3.39 ± 1.95
Homoserine, threonine	−6.80 ± 5.20
Hydantoin-5-propionic acid	−2.14 ± 0.72
Diphosphoinositol pentakisphosphate	−2.35 ± 1.02
Myo-inositol hexakisphosphate	−2.54 ± 1.21
Leukotriene D4	14.54 ± 5.01
Leukotriene E4	−4.21 ± 1.67
Xanthine	39.62 ± 14.12
Guanosine	12.71 ± 9.35
Inosine	3.83 ± 2.20
Cytidine	2.67 ± 1.22
Nicotinic acid mononucleotide	−5.93 ± 2.50
Nicotinic acid	2.30 ± 0.93
Niacinamide	−2.13 ± 0.62
Pantothenic acid	−3.17 ± 1.59
2-Aminomuconic acid semialdehyde	1.96 ± 0.68

The p-values were calculated using ANOVA followed by a Tukey test and the fold changes were calculated using Fieller's theorem for a 95% confidence interval. A positive fold change indicates a higher level in 74A UM-SCC-74A as compared to 74B UM-SCC-74B

In the radiosensitive cell line UM-SCC-74A, very few metabolic alterations were observed after irradiation (Table 2). The main metabolic alterations were linked to tryptophan metabolism through tryptophan and 5-hydroxyindoleacetic acid (5-HIAA) (*p* = 0.0006). In contrast, the less radiosensitive cell line UM-SCC-74B showed numerous metabolic alterations after irradiation, where the main metabolic alterations were connected to nicotinate and nicotinamide metabolism (*p* = 0.003), the methionine cycle (*p* = 0.05), and purine metabolism (*p* = 0.001) (Table 3). A number of metabolites involved in the nicotinate and nicotinamide metabolism such as 1-methylnicotinamide, niacinamide, beta-nicotinamide D-ribonucleotide, nicotinic acid mononucleotide, and nicotinamide adenine dinucleotide (NAD) were all down-regulated in the UM-SCC-74B 24 h after irradiation. Several metabolites involved in the methionine cycle as well as the glycine and serine biosynthesis and metabolism were found altered in the irradiated UM-SCC-74B cells. Increased levels of methionine were observed 4 h after irradiation, while the levels of S-adenosylmethionine (SAM) was increased both 4 h and 24 h after irradiation. Metabolites involved in purine metabolism such as adenosine, guanosine, and guanine were upregulated 24 h after irradiation while guanosine mono phosphate (GMP) was found in significantly higher levels 4 h after irradiation as compared to 24 h after irradiation.

**Table 2:** Significantly altered metabolites (*p* < 0.05) in the cell line UM-SCC-74A between cells irradiated with 2 Gy [assessed 4 h (rapid) or 24 h (intermediate) after irradiation] vs. non-irradiated cells (control) from the analysis in negative ionization mode are presented as fold changes (95% confidence intervals).

**Metabolite**	**Rapid vs. control**	**Intermediate vs. control**	**Rapid vs. intermediate**
**NEGATIVE IONIZATION**			
Phenylalanine		1.84 ± 0.62	
Uridine	−2.27 ± 0.88	1.62 ± 0.55	−3.54 ± 1.51
Adenosine			3.94 ± 2.20
2-Aminomuconic acid semialdehyde			−1.54 ± 0.51
5-Hydroxyindoleacetic acid		2.26 ± 1.10	−2.49 ± 1.28
**POSITIVE IONIZATION**			
Tryptophan	2.23 ± 0.94		2.48 ± 1.15

*The p-values were calculated using ANOVA followed by a Tukey test and the fold changes were calculated using Fieller's theorem for a 95% confidence interval. A positive fold change indicates a higher level in the radiated cells as compared to the control*.

**Table 3:** Significantly altered metabolites (*p* < 0.05) in the cell line UM-SCC-74B between cells irradiated with 2 Gy [assessed 4 h (rapid) or 24 h (intermediate) after irradiation] vs. non-irradiated cells (control) from the analysis in positive ionization mode are presented as fold changes (95% confidence).

**Metabolite**	**Rapid vs. control**	**Intermediate vs. control**	**Rapid vs. intermediate**
**POSITIVE IONIZATION**			
Phenylalanine	2.18 ± 1.18		3.51 ± 2.18
Proline	2.64 ± 1.47		3.72 ± 2.12
Tyrosine	3.89 ± 2.63		8.32 ± 6.08
Glutamine			2.62 ± 1.35
Methionine	2.94 ± 1.27		3.68 ± 2.23
Valine	2.70 ± 1.74		5.07 ± 3.53
Taurine	2.93 ± 1.38		5.20 ± 2.87
Alanine, beta-alanine, sarcosine			1.60 ± 0.43
Creatine	4.14 ± 2.81		7.64 ± 5.24
Glucosamine, fructosamine			4.02 ± 2.89
N-Acetyl-glucosamine-1-phosphate	2.27 ± 1.23		3.90 ± 2.50
Glucosamine-1-phosphate	2.06 ± 0.75		4.90 ± 3.28
Dihydroneopterin triphosphate		1.58 ± 0.37	
Glycerophosphocholine	5.73 ± 4.13		10.56 ± 8.03
Hydroxyindole	1.71 ± 0.70		2.20 ± 0.96
Dityrosine			2.43 ± 1.30
Thiamine triphosphate	2.26 ± 1.19		3.81 ± 2.36
1-Methylnicotinamide			2.01 ± 0.88
Pantothenic acid			8.52 ± 7.61
UMP			3.35 ± 1.94
**NEGATIVE IONIZATION**			
Tyrosine		−9.01 ± 2.63	7.81 ± 3.92
Arginine		−4.37 ± 1.92	
Proline		−17.16 ± 7.94	
Valine		−23.50 ± 8.91	
Taurine		−13.71 ± 6.30	
Alanine, beta-alanine, sarcosine		−28.73 ± 23.88	
Homoserine, threonine		−39.49 ± 34.97	
L-Dopa	−3.78 ± 2.15		
Choline		−5.81 ± 2.33	5.06 ± 3.08
Docosanaoyl-CoA		45.98 ± 21.19	
Linolenoyl-CoA		−13.44 ± 11.25	
Oleoyl-CoA		−7.94 ± 4.77	
S-Adenosylmethionine	5.19 ± 3.36	4.74 ± 2.96	
Hydantoin-5-propionic acid		−6.50 ± 2.61	6.20 ± 3.38
1-Arachidonoylglycerophosphoinositol		3.74 ± 2.01	
Diphosphoinositol pentakisphosphate		−16.08 ± 3.31	
Myo-inositol hexakisphosphate		−7.94 ± 2.88	
Leukotriene E4		−24.29 ± 14.42	5.55 ± 0.38
Guanine		6.21 ± 2.41	−20.50 ± 19.17
Adenosine		3.59 ± 2.52	
Guanosine		19.83 ± 18.36	
Pantothenic acid		−19.21 ± 7.12	
Nicotinic acid mononucleotide		−8.92 ± 3.90	7.56 ± 5.20
1-methylnicotinamide		−6.75 ± 1.87	
Niacinamide		−6.34 ± 1.97	5.96 ± 2.41
beta-nicotinamide D-ribonucleotide		−5.11 ± 1.59	6.86 ± 4.32
NAD		−6.50 ± 2.79	8.18 ± 5.27
CMP			21.64 ± 19.67
GMP			17.49 ± 15.52

*The p-values were calculated using ANOVA followed by a Tukey test and the fold changes were calculated using Fieller's theorem for a 95% confidence interval. A positive fold change indicates a higher level in the radiated cells as compared to the control*.

## Discussion

A current clinical problem in HNSCC is the varying therapeutic response to irradiation, from complete to no response, moreover relapse and resistance is common (64). Individualized dosimetry, e.g., by predicting or monitoring tumor radiation response before, during, or after treatment, could help optimize radiotherapy, improve therapeutic outcome, and reduce normal tissue complication after radiotherapy. The plasticity of cancer cell metabolism plays a major role in cancer cell survival and treatment-resistant disease. Cancer cells are able to adapt metabolically to many types of cellular stress, such as radiation, and previous studies have demonstrated alterations in cellular metabolites after irradiation of the cells (40, 41, 43, 65–68). Consequently, a feasible way to provide significant understanding on the mechanisms of radiation response may be through metabolic profiling. In the present study, we have assessed metabolic profiling as a mean to investigate the relationship between radiation response and the metabolome. This was done by utilizing a unique *in vitro* model, in which two HNSCC cell lines from the same donor were employed. These cells exhibited the same STR genetic profile (Supplemental Table 1) but were shown to display different radiosensitivities, where UM-SCC-74A was shown to be clearly more sensitive to radiation than UM-SCC-74B in four independent assays (Figures 1, 2, Supplemental Figure 1). These assays reflect different thresholds, parameters, and time-frames for assessment of cell viability and growth. Consequently, they are to be seen as important complements to each other in order to assess the full spectrum of radiation response.

### UM-SCC-74B Cells Were More Radioresistant Than UM-SCC-74A Cells

In the clonogenic survival assay, considered as a gold standard in radiation research (48, 69), UM-SCC-74A displayed a lower survival fraction at all radiation doses assessed, at 2 Gy approximately half of that of UM-SCC-74B (Figures 1A,B). The assay combines contribution of all types of cell death, encompassing both early and late events. However, the impact of cell-to-cell communication is disregarded. Moreover, quiescent cells and cells growing at a slower rate may be counted as non-surviving clones in the clonogenic survival assay.

In contrast, the long-term growth inhibition assay, and in particular the 3D cell culture assay, include cell-to-cell communication, and both assays reflect both cell death and cell growth inhibition. The long-term growth inhibition assay is an especially suitable complement to clonogenic survival assays at higher doses, where the plating efficiency for clonogenic survival may be too low to ensure reliable results. The longer time-frame also enables visualization of delayed or reduced growth. This was demonstrated in the present study, e.g., for the 8 Gy irradiated samples, where UM-SCC-74A cells resumed growth after 3 weeks, whereas, UM-SCC-74B growth was resumed already after 2 weeks (Figures 2A,B). Results in the long-term growth inhibition assay (Figure 2) verified the clear differences in radiosensitivity at higher doses observed in the clonogenic survival assays (Figure 1A). Also at 2 Gy, UM-SCC-74A cells demonstrated lower cell growth and viability than UM-SCC-74B cells, although not as pronounced as in the clonogenic survival assay, potentially reflecting the different time-frames, culture conditions, and assessed parameters in the different assays. The long-term growth assay also visualized the different doubling times of the cell lines, where UM-SCC-74B demonstrated a shorter doubling time than UM-SCC-74A. This is consistent with clinical experiences, where the recurrence of a tumor is often faster growing and more aggressive. The differences in growth rates were also in line with the clearly separated PCA scores plot (Figure 3), and reflected in some of the metabolic differences between the unirradiated cell lines (Table 1), discussed more in detail below. The fact that the STR profiles were the same, whereas factors such as growth rates, radiation resistance, and metabolic profiles were not, also demonstrates the advantage of complementing genetic data with cell assays and metabolomic profiling.

The 3D cell culture assay visualizes radiation effects on viability and cell growth in the same time frame as clonogenic survival. However, in the 3D assay, cells are cultured in a more *in vivo* like situation. In addition to cell-to-cell communication, important factors such as oxygen and nutritient gradients are mimicking the environment of small non-vascularized metastases (63). Results from the 3D cell culture assay were in line with the clonogenic survival assay, where both assays demonstrated approximately twice as many surviving UM-SCC-74B cells than UM-SCC-74A cells 10–14 days after 2 Gy irradiation (Figures 1B,C), further validating the difference in radiosensitivity between the cell lines. Consequently, we conclude that in all three independent assays UM-SCC-74A cells were clearly more affected by radiation in terms of cell viability and growth than UM-SCC-74B cells. This was also in line with the apoptosis assay, demonstrating increased levels of cleaved PARP1 in irradiated UM-SCC74A cells, but not in UM-SCC-74B cells (Supplemental Figure 1). Thus, we concluded that the cell lines were a suitable model system for subsequent complex metabolic evaluations of radiation response.

### Unirradiated Cells Differed in Nicotinamide and Nicotinic Acid Metabolism and Purine Metabolism Pathways

**Figure 4:**
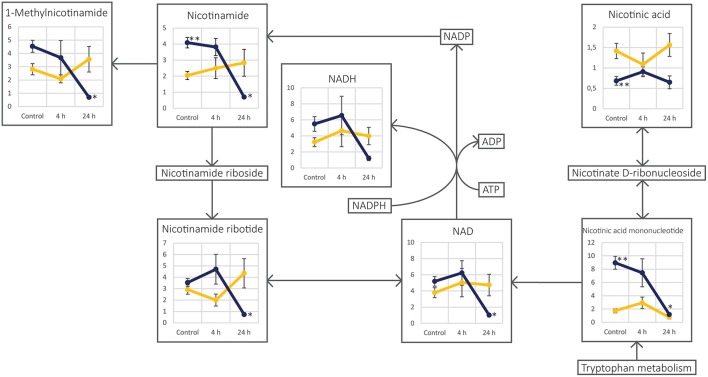
Schematic figure of the nicotinamide and nicotinic acid metabolism pathway in the two cell lines UM-SCC-74A (yellow lines) and UM-SCC-74B (dark blue lines). Each annotated metabolite is represented by a line-plot with normalized peak area on the y-axis and the three different sample groups; Control, 4 and 24 h after irradiation, on the x-axis. Error bars represent standard error of the mean. *Metabolite level was significantly (*p* < 0.05) altered as compared to the respective control group. **Metabolite level was found significantly different (*p* < 0.05) between the two cell line control groups.

Even though unirradiated UM-SCC-74A and UM-SCC-74B cells were identical according to STR genotyping, metabolic profiles differed clearly between the two unirradiated cell lines, as they were distinctly separated in the PCA scores plot (Figure 3). The nicotinamide and nicotinic acid metabolism pathways were indicated as important differences in the pathway analysis between the two cell lines, with metabolites such as nicotinamide, nicotinic acid, and nicotinic acid mononucleotide significantly different between the two control groups (Figure 4, Table 1). Nicotinamide and nicotinic acid mononucleotide were found at significantly higher levels in unirradiated UM-SCC-74B cells compared to UM-SCC-74A cells, while nicotinic acid was found in higher levels in unirradiated UM-SCC-74A cells compared to UM-SCC-74B cells (Figure 4). This indicates a lower rate of biosynthesis of nicotinic acid mononucleotide and ultimately in nicotinamide adenosine dinucleotide (NAD+) in the UM-SCC-74A cell line. The other main pathway that was found differentiating between the two non-irradiated cell lines was purine metabolism. Xanthine, inosine, and guanosine were all found at significantly higher levels in the UM-SCC-74A cells than in the UM-SCC-74B cells. The higher levels of inosine and xanthine in UM-SCC-74A may indicate a higher purine degradation in the UM-SCC-74A cell line. In UM-SCC-74B, 5-phosphoribosylamine was found in higher concentration, which is an intermediate in *de novo* purine synthesis, and might indicate a higher rate of *de novo* synthesis of purine nucleotides in the UM-SCC-74B cells. Higher rates of purine *de novo* synthesis have previously been linked to increased growth rates (70), and is in line with the results from the growth inhibition assay, where unirradiated UM-SCC-74B cells demonstrated a shorter doubling time than UM-SCC-74A cells (Figure 2).

### Altered Tryptophan and Serotonin Metabolism in Irradiated UM-SCC-74A Cells

In general, very few metabolic alterations were observed after irradiation in UM-SCC-74A cells compared to UM-SCC-74B cells. In the UM-SCC-74A cell line the most important radiation induced metabolic perturbation was the alteration in tryptophan metabolism. Tryptophan was found at higher levels as compared to the control 4 h after radiation but was reduced to the same levels as the control 24 h after radiation. Tryptophan has two main metabolic fates, it can be used in the biosynthesis of quinolinic acid, which is a precursor to nicotinic acid mononucleotide, or it can be converted to serotonin via 5-hydroxytryptophan. The increase in tryptophan levels 4 h after irradiation could be an indication of a shift in the metabolism toward an increase in quinolinic acid and nicotinic acid mononucleotide biosynthesis in the UM-SCC-74A cell line. However, this assumption could not be verified, as the nicotinic acid mononucleotide levels were not altered neither 4, nor 24 h after irradiation in UM-SCC-74A, and quinolinic acid was not detected in this analysis. Moreover, the serotonin metabolite 5-hydroxyindoleacetic acid (5-HIIA) was found increased 24 h after irradiation the UM-SCC-74A cell line, which might suggest an increase in serotonin degradation to 5-HIIA. Serotonin has been shown to exhibit growth stimulatory effects on several types of carcinoma and other tumor cells, and to play a role in radiation-induced bystander effect (71). In previous studies, serotonin concentrations in culture media have been shown to be depleted after exposure of cells to radiation, suggesting that serotonin may be bound by membrane receptors after irradiation, thus facilitating calcium entry into cells, production of ROS and activation of apoptosis pathways (71, 72). Serotonin can also act as both vasoconstrictor and promote angiogenesis in solid tumors, and could therefore have important effects on the tumor hypoxia which have been linked to radiation sensitivity previously (73).

### Decreased Levels of NAD+ and Increased NAD+ Turnover Suggest Initiated DNA Repair Mechanisms in Irradiated UM-SCC-74B Cells

In the UM-SCC-74A cell line, no investigated metabolites in the nicotinamide and nicotinic acid metabolism demonstrated significantly altered levels after irradiation. This is in contrast to the UM-SCC-74B cell line, where the metabolites nicotinamide, 1-methylnicotinamide, nicotinamide ribotide, nicotinic acid mononucleotide, NADH as well as NAD+ were all almost depleted 24 h after irradiation (Figure 4). This demonstrates inherent differences in nicotinamide and nicotinate metabolism in the two cell lines both before and after irradiation. While the decrease in NAD+ could be due to an increase in turnover from NAD+ to NADP/NADPH to maintain the redox status in the cells, regenerate glutathione, and an increased biosynthesis (45), the fact that NADP+ and NADPH were not detected in the analysis contradicts this explanation. Thus, a more likely explanation is that the decreased levels of NAD+ and increased NAD+ turnover in UM-SCC-74B cells after irradiation is due to an increased ADP-ribosylation by poly(ADP-ribose)polymerases (PARPs) to initiate DNA repair mechanisms (74–77). The PARP proteins are the main consumers of NAD+ during genotoxic stress, and the levels of NAD+ can be depleted following ionizing radiation to meet the demands for DNA-repair signaling by ADP-ribosyl (74–76). PARP1 is a member of the PARP family of enzymes. Its primary function is to detect and repair DNA damage, where amplified PARP1 activity results in high NAD+ consumption. This process is blocked by rapid cleavage and inactivation of PARP1 by the action of caspases. In the present study, the cleaved PARP1 assay demonstrated that the levels of inactivated PARP1 were increased in irradiated UM-SCC-74A cells, whereas levels in UM-SCC-74B cells were unchanged after radiation (Supplemental Figure 1), which could further support this hypothesis. Moreover, previous studies have demonstrated that inhibition of PARP or PARP silencing increase radiosensitivity (78–85). Van Vuurden et al. (81) observed an overexpression of PARP1 as well as a radiosensitizing effect by the PARP1 inhibitor olaparib in pediatric medulloblastoma, ependymoma, and high grade glioma cell lines. Similarly, Owonikoko et al. (84) investigated the PARP1 inhibitor veliparib in combination with DNA-damaging treatments including radiation in small cell lung cancer cells, and found that veliparib sensitized some cells to DNA damaging treatment. Both Godon et al. (80) and Noël et al. (82) found that the radiosensitizing effect of PARP1 inhibition by 4-amino-1,8-naphthalimide (ANI) was cell cycle dependent, and that rapidly growing cells with high fraction of cells in the S-phase were more sensitive to PARP-inhibition in combination with radiation. Consequently, our data suggest that PARP inhibition may be especially suitable to overcome the radioresistance in the radioresistant UM-SCC-74B cells, and should be evaluated in future studies.

### Increased Levels of Guanosine and Adenosine Indicate a Functioning Purine Salvage Pathway and More Efficient ROS Protection in Irradiated UM-SCC-74B Cells

In UM-SCC-74B, the levels of adenosine, guanosine and guanine were increased 24 h after irradiation, while the levels of adenosine monophosphate (AMP) and guanosine monophosphate (GMP) were decreased (not significantly) 24 h after irradiation. This indicates a functioning purine salvage pathway with an increased degradation of the purine nucleotides to the corresponding nucleosides and nucleobases. This is in line with previous studies, where guanosine have been shown to protect DNA *in vitro* from oxidative damage induced by reactive oxygen species (ROS), and to serve as a radioprotector (86). Adenosine has demonstrated the same effect as guanosine however not as strong, while the pyrimidine nucleobases had the opposite effect (86). In contrast, this pattern was not observed in the UM-SCC-74A cell line, where altered (although not significant) levels of inosine, hypoxanthine, and xanthine instead might indicate an increased purine nucleotide catabolism through the transformation of inosine to hypoxanthine to xanthine. This data suggests that the UM-SCC-74B cells may have enabled a more efficient ROS protection through increased levels of guanosine and adenosine after irradiation.

### Increased Levels of SAM Indicate Alterations in the DNA Methylation in Irradiated UM-SCC-74B Cells

**Figure 5:**
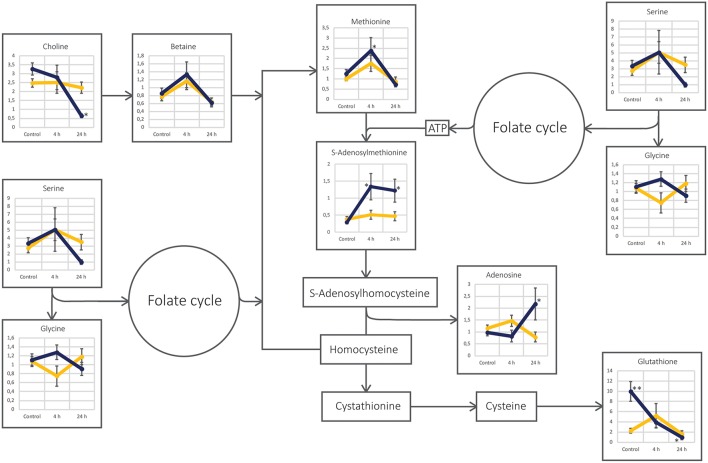
Schematic figure of the cysteine and methionine metabolism pathway in the two cell lines UM-SCC-74A (yellow line) and UM-SCC-74B (dark blue line). Each annotated metabolite is represented by a line-plot with normalized peak area on the y-axis and the three different sample groups; Control, 4 and 24 h after irradiation, on the x-axis. Error bars represent standard error of the mean. *Metabolite level was significantly (*p* < 0.05) altered as compared to the respective control group. **Metabolite level was found significantly different (*p* < 0.05) between the two cell line control groups.

The pathway of cysteine and methionine metabolism was altered in the UM-SCC-74B cell line after irradiation, in contrast to UM-SCC-74A cells (Figure 5). S-adenosylmethionine (SAM) is a methyl donor, involved in almost all methylation reactions in the cells, such as DNA and histone methylation but also methyl transfer reactions to proteins, lipids, and secondary metabolites (3, 4). SAM is also an important component in many trans-sulfuration reactions and aminopropylation reactions (87). After irradiation, there was an increase in both methionine and SAM in UM-SCC-74B cells, suggesting an increased turnover from homocysteine to methionine, which can be driven by either the folate cycle or methyl-group transfer by betaine (87). As the levels of glycine and serine were not found altered after irradiation and neither were the levels of 5-methyltetrahydrofolate (data not shown), data suggest that the turnover from homocysteine to methionine was not driven by the folate cycle but rather betaine (88). This was supported by the decreased levels on choline, the main precursor of betaine, 24 h after irradiation. Moreover, the levels of glutathione in UM-SCC-74B cells were lower after irradiation, indicating that homocysteine is not converted to cystathionine, but mainly reconverted to methionine, since cysteine is the rate-limiting precursor for biosynthesis of glutathione (87). Consequently, our data suggest that irradiated UM-SCC-74B cells mobilized the homocysteine-methionine cycle, thereby increasing the synthesis of SAM to avoid radiation induced DNA-hypomethylation (89). This is in line with previous studies, linking global changes in DNA methylation to ionizing radiation (90–93), and to the development of radioresistance in oral squamous cell carcinoma (93) and lung cancer (92). Kim et al. (92) found that several key regulators in radiosensitivity in lung cancer were epigenetically controlled by CpG methylation. Batra et al. (94) demonstrated that methyl donor deficient diets increased the irradiation induced metabolic stress in mice and decreased DNA methyl transferase activity, indicating decreased DNA methylation (94, 95). Moreover, Batra et al. (96) demonstrated that L-methionine supplementation might help to alleviate radiation induced loss of genomic DNA methylation in murine liver tissue. This could open up for new possibilities of sensitizing tumors to radiation treatment and in the future avoid radio-resistance in radiation treatment.

## Conclusion

Our data strongly implicates that the radioresistant cells changed their metabolism to control the redox status, DNA repair as well as DNA methylation. A lot of preclinical efforts have over time been devoted to the development of strategies to sensitize cancer cells to radiation therapy. This study was a first step in the understanding of which metabolic pathways in SCC that were important for the differences in radiosensitivity between the two cell lines UM-SCC-74A and UM-SCC-74B. The elucidation of the mechanisms behind radioresistance could lead to better prediction of radiation treatment outcome or possibilities to sensitize tumors to radiation. However, all metabolites and metabolic pathways investigated in this study all require further investigation as to whether they will be able to pose as targets for prediction of radiation response or to enhance radiation sensitivity.

## Data Availability

The datasets for this study are available on request.

## Author Contributions

EL, IE, ME, JH, TA, CP, GL, and MN designed the study. EL, IE, ME, JH, TA, MH, CP, GL, and MN contributed to data analysis and interpretation, and revised the manuscript. EL and IE contributed to experimental studies and drafted the manuscript.

### Conflict of Interest Statement

The authors declare that the research was conducted in the absence of any commercial or financial relationships that could be construed as a potential conflict of interest.

## Supplementary Material

Supplemental Figure 1 Levels of cleaved PARP1 in UM-SCC-74A (black bars) and UM-SCC-74B (gray bars) cells 12 h after 0 or 2 Gy irradiation, measured by ELISA. Error bars represent the standard error of mean *N* = 3.

Supplemental Table 1 STR analysis of non-irradiated UM-SCC-74A and UM-SCC-74B.
