# Supplementary figures and images for: Exploring Radiation Response in Two Head and Neck Squamous Carcinoma Cell Lines Through Metabolic Profiling
Source: Front Oncol. 2019 Aug 30;9:825. doi: 10.3389/fonc.2019.00825 (PMC6728927; doi:10.3389/fonc.2019.00825)

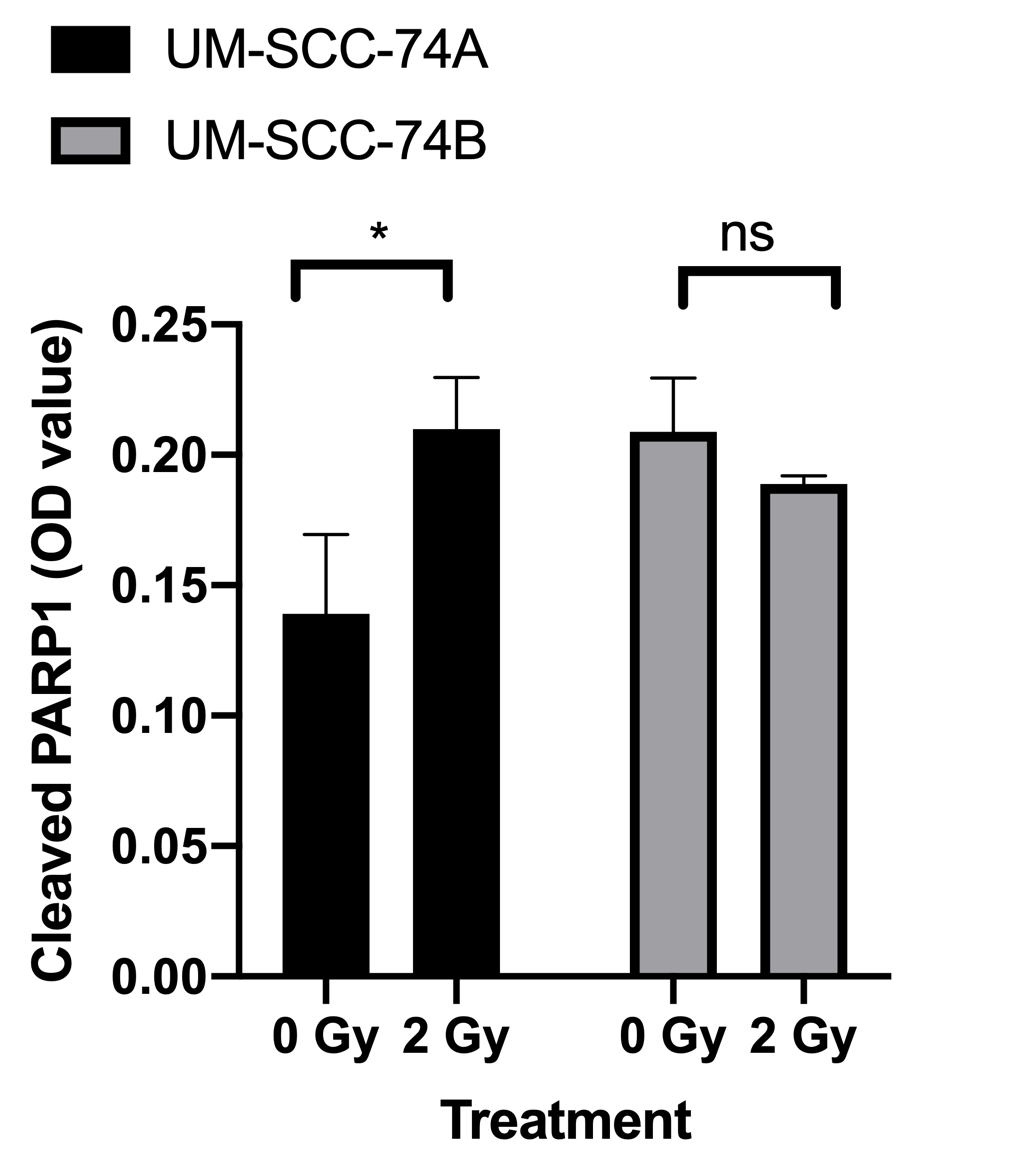

Supplement: Supplemental Figure 1 — Levels of cleaved PARP1 in UM-SCC-74A (black bars) and UM-SCC-74B (gray bars) cells 12 h after 0 or 2 Gy irradiation, measured by ELISA. Error bars represent the standard error of mean N = 3. [file Image_1.TIFF]
