# Supplementary material for: Exploring Radiation Response in Two Head and Neck Squamous Carcinoma Cell Lines Through Metabolic Profiling
Source: Front Oncol. 2019 Aug 30;9:825. doi: 10.3389/fonc.2019.00825 (PMC6728927; doi:10.3389/fonc.2019.00825)
Supplement: Supplemental Table 1 — STR analysis of non-irradiated UM-SCC-74A and UM-SCC-74B. [file Table_1.DOCX]

*Table 1. STR analysis of UM-SCC-74A and UM-SCC-74B*

| **Sample marker** | **UM-SCC-74A** | | **UM-SCC-74B** | |
| --- | --- | --- | --- | --- |
|  | Allele 1 | Allele 2 | Allele 1 | Allele 2 |
| **Amelogenin** | X | X | X | X |
| **CS1FPO** | 9 | 12 | 9 | 12 |
| **D13S317** | 12 | 12 | 12 | 12 |
| **D16S539** | 10 | 12 | 10 | 12 |
| **D18S51** | 17 | 17 | 17 | 17 |
| **D19S433** | 14 | 16,2 | 14 | 16,2 |
| **D21S11** | 30 | 34,2 | 30 | 34,2 |
| **D2S1338** | 20 | 20 | 20 | 20 |
| **D3S1358** | 15 | 16 | 15 | 16 |
| **D5S818** | 12 | 12 | 12 | 12 |
| **D7S820** | 11 | 11 | 11 | 11 |
| **D8S1179** | 12 | 13 | 12 | 13 |
| **FGA** | 21 | 26 | 21 | 26 |
| **TH01** | 6 | 9,3 | 6 | 9,3 |
| **TPOX** | 8 | 8 | 8 | 8 |
| **vWA** | 15 | 16 | 15 | 16 |
